# A novel 3D insect detection and monitoring system in plants based on deep learning

**DOI:** 10.3389/fpls.2023.1236154

**Published:** 2023-08-31

**Authors:** Nak Jung Choi, Kibon Ku, Sheikh Mansoor, Yong Suk Chung, Thai Thanh Tuan

**Affiliations:** ^1^ Crop Foundation Division, National Institute of Crop Science, Rural Development Administration, Jeollabuk-do, Republic of Korea; ^2^ Department of Plant Resources and Environment, Jeju National University, Jeju, Republic of Korea

**Keywords:** insect, 3D reconstruction, behavior analysis, monitoring, habit exploration

## Abstract

Insects can have a significant impact on biodiversity, ecology, and the economy. Certain insects, such as aphids, caterpillars, and beetles, feed on plant tissues, including leaves, stems, and fruits. They can cause direct damage by chewing on the plant parts, resulting in holes, defoliation, or stunted growth. This can weaken the plant and affect its overall health and productivity. Therefore, the aim of this research was to develop a model system that can identify insects and track their behavior, movement, size, and habits. We successfully built a 3D monitoring system that can track insects over time, facilitating the exploration of their habits and interactions with plants and crops. This technique can assist researchers in comprehending insect behavior and ecology, and it can be beneficial for further research in these areas.

## 1 Introduction

The issue of biological invasion of insects has become a significant focus in ecological research in recent years. One critical aspect of studying invasive species is monitoring their growth and development. Invasive insect herbivores cause an estimated global loss of at least 70 billion USD annually, with countries like the USA and China experiencing the highest costs and also serving as major sources of invasive pests (Bradshaw et al., 2016; Paini et al., 2016). The economic impact of invasive insects is increasing due to factors like globalization and climate change. Herbivorous stink bugs (Hemiptera: Pentatomidae) are a prime example of this, with many species acting as agricultural pests in their native regions and causing significant economic damage when they invade new areas. Stink bugs harm crops by puncturing plant tissues, especially fruits and seeds, which can lead to reduced yield and quality. Some species also transmit plant pathogens (Schaefer and Panizzi, 2000; Paini et al., 2016; McPherson, 2018; Insectaeva et al., 2020).

In arable crops, major insect pests that cause significant economic impact are those that can quickly infest large areas, including hemipterans, beetles, thrips, and flies. These pests tend to cluster in certain areas of fields rather than being evenly distributed. To effectively address infestations, it is important to know the location and size of the affected areas. However, because insects can rapidly spread throughout a field during favorable weather conditions, quick action is necessary (Li and Yang, 2020; Bieganowski et al., 2021). Traditional methods of visually assessing field health along a dense sampling grid are not practical due to the time and labor involved, making it difficult to generate pest maps and precise spraying maps. Additionally, identifying insects that are beneath the crop canopy or on the undersides of leaves can be challenging. Insect nets, commonly used in research, provide an option for capturing insect from the crop to aid in identification and removal (Li and Yang, 2020; Bieganowski et al., 2021).

To improve insect detection and monitoring, researchers are working on developing tools for automated image analysis that can recognize insects under various weather and illumination conditions, as well as on different types of crops with varying leaf colors and shapes. While progress has been made in this area, artificial intelligence and machine learning advancements in image analysis are expected to bring even greater success in creating automated sensors for precise insect pest control in the future (McCravy, 2018). Real-time analysis during the scanning process can save time by automatically detecting insects, leading to efforts to develop small systems that combine cameras and computers into one intelligent sensor unit. These embedded systems are becoming more affordable and faster, allowing for the use of large, deep-learning models to recognize insect with greater accuracy. Various techniques, including visual inspection, suction traps, and passive methods, can be used to monitor both insect pests and beneficial. Insect pest camera-based monitoring is a practical approach that can be used in agriculture and forestry, complementing aerial and field surveys for pests. Remote sensing, which uses images from satellites or unmanned aerial vehicles such as drones, can also be used to create infestation maps, along with traditional aerial and field scouting methods. Camera traps for insect pests, combined with remote sensing, can improve monitoring programs in forests by allowing for early detection of harmful species and prediction of outbreak risks based on monitoring data. These monitoring improvements can also be applied to the monitoring of quarantine pests (Ayres and Lombardero, 2018; Choi and Park, 2019; Zhang et al., 2019).

The main objective of insect pest monitoring within integrated pest management programs in agriculture is to provide growers with a useful decision-making tool. This is achieved through the establishment of intervention thresholds to address insect pest infestations at the most opportune time, optimizing control strategies and minimizing grower inputs on that particular crop (Dent, 2000). The data collected from monitoring can also be used to develop prediction phenological models to forecast insect population outbreaks, providing additional information to improve control techniques and optimize insecticide usage. In forestry, detecting and monitoring both native insect pests and invasive species are critical in developing appropriate management programs. Forest insect species can have a significant impact on the biodiversity, ecology, and economy of the affected area, making effective monitoring and control essential (Dent, 2000; Brockerhoff et al., 2006).

Various monitoring methods are available for determining the extent of crop damage caused by insects within a field, including the use of spectrometers, true-color cameras, multi- and hyperspectral cameras, and stereo camera systems. Three-dimensional (3D) information can be obtained from UAV camera flights using structure from motion algorithms which is the process of estimating the 3D structure of a scene from a set of 2D images. This 3D information can provide the details about the shape and movement of insect. Monitoring and sampling insects can provide valuable insights into the behaviors of different species, as well as their roles in pollinating crops. However, conventional methods of insect monitoring are labor-intensive and time-consuming.

## 2 Materials and methods

There are insects on a plant that is enclosed in a box. Two precisely aligned cameras in video recording devices are positioned to observe consecutive frames of insect movements and record into a data storage system. Insect detectors are employed to identify the locations of the insect on each frame. With the information of the system setup, such as position of the cameras and distance between the cameras and plant, and the insect position in the frame, it is possible to estimate the insects’ 3D location in world coordinates for that particular frame. The 3D location is stored into a point cloud file. Then, an open library mpl_toolkits.mplot3d (‘The mplot3d toolkit.’, 2023) is used to visualize insects in 3D world coordinates. Those insects’ 3D locations over frames of the videos are further analyzed to obtain the moving speed and moving route of the insects. This information is valuable for analyzing the habitat and insect behavior patterns.

### 2.1 System specifications

**Figure 1:**
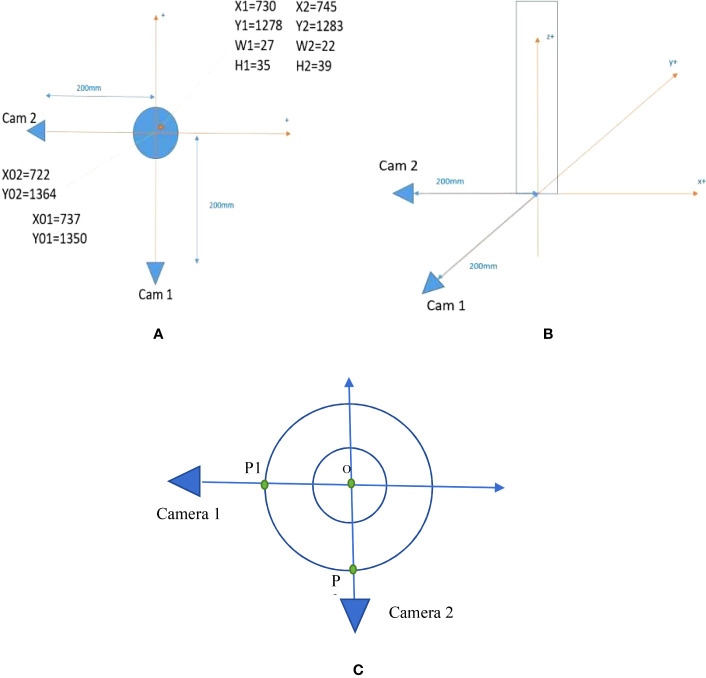
Setup of the camera system. **(A)** Top view of the system. **(B)** Side view of the system. **(C)** The setup to line up the camera orientations precisely.

Two cameras are used in this system to build a 3D representation of the insects’ positions and movements. **Figure 1** shows the setup of the system. We set the coordinate system at the center of the planting pot. The positive direction of *x*-axis, *y*-axis, and *z*-axis are shown in **Figure 1B** with orange arrows.

**Figure 2:**
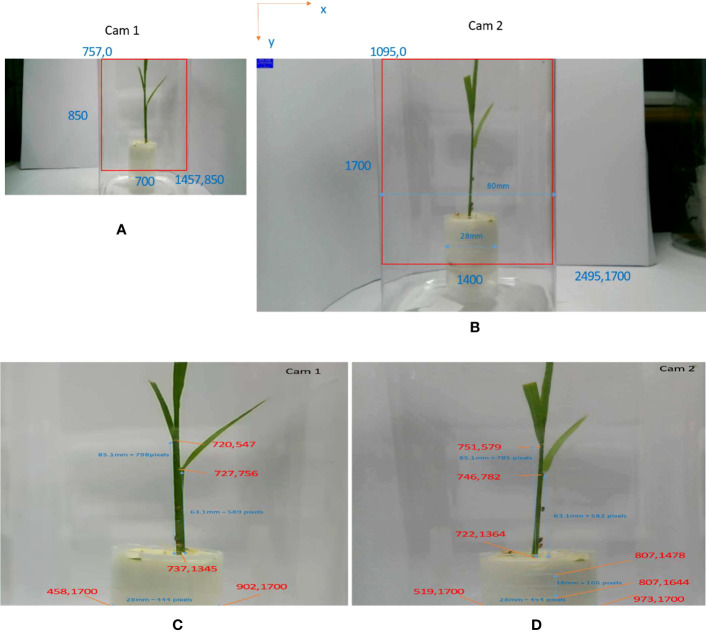
System setup information. **(A)** Cropped region in the first camera. **(B)** Cropped region in the second camera. **(C)** Region of interest from camera 1 and some special points. **(D)** Region of interest from camera 2 and some special points.

**Figure 3:**
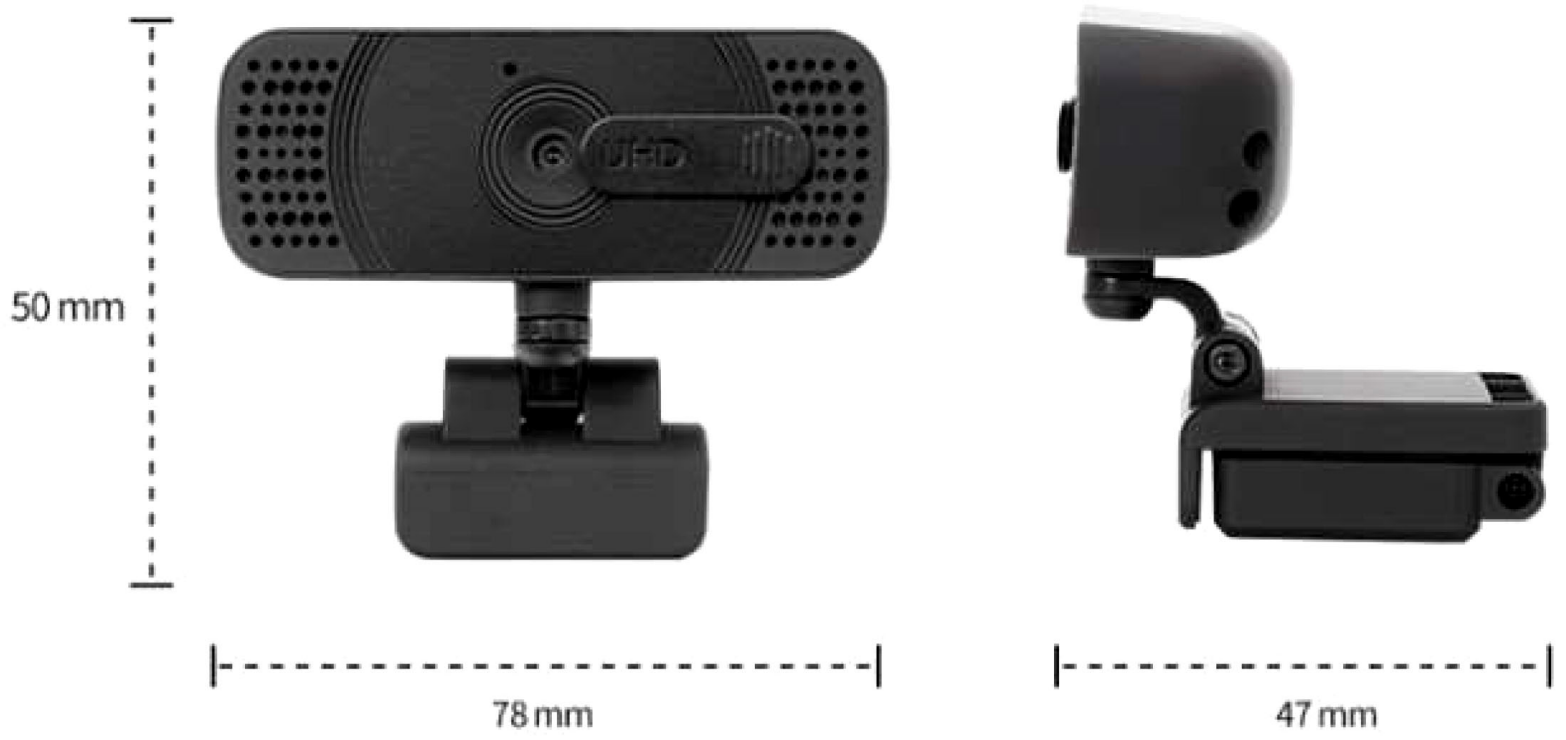
The cameras used in this research.

The two cameras are placed 200 mm far from the center of the planting pot. The camera is strategically positioned to enable comprehensive monitoring of the plant, as visually represented in **Figure 2**. This intentional placement ensures that the plant is optimally centered horizontally within the image frame, thereby facilitating efficient and effective monitoring and observation. The origin of coordinates is set at (*X*_01 =_ 737, *Y*_01 =_ 1,350) pixels in the first camera (Cam 1) cropped image and (*X*_02 =_ 722, *Y*_02 =_ 1,364) pixels in the second camera (Cam 2) cropped image. One example of an insect appears in **Figure 1A** at the location (*X*_1 =_ 730, *Y*_1 =_ 1278) pixels with bounding box (*W*_1 =_ 27, *H*_1 =_ 35) pixels in the Cam1 cropped image and (*X*_2 =_ 745, *Y*_2 =_ 1,283) pixels with bounding box (*W*_2 =_ 22, *H*_2 =_ 39) pixels in the Cam2 cropped image. *W*_1_, *W*_2_/*H*_1_, *H*_2_ are the width/height of the bounding boxes. The width of the planting pot is 28 mm and the cameras are placed at 86 mm height from the ground. **Figure 3** shows the image of the camera that is used in this research, HW40 4K UHD Hansung autofocus. Because the insects are small, we choose a camera with high resolution for getting a clear video of the insects. These cameras provide wide-angle cameras of 77°; thus, we can place them near the plant and the insect.

One plant is placed in the center of the planting pot. Three insects are kept on the plant for monitoring. We record videos from the two cameras. These videos are extracted to get frame images that are used for training the detector. We choose one video (approximately 18 min long) for testing the system. We extract the region of interest (ROI) from images from camera 1 and camera 2. The image from the first camera in **Figure 2A** was cropped by a bounding box with the top left corner at (757,0) and the bottom right corner at (1457,850). Then the cropped image is resized with bilinear interpolation to the size (1400,1700) in ROI1, **Figure 2C**. The image from the second camera in **Figure 2B** was cropped by a bounding box with the top left at (1095,0) and the bottom right at (2495,1700). The bounding box was used as a region of interest (ROI2) with the same size as ROI1.

To ensure precise orientation determination, a deliberate methodology is employed, involving the placement of two distinct points, designated as P1 and P2 in **Figure 1C**, along a circular trajectory. The fundamental requirement is that the angle formed between the vectors OP1 and OP2 measures exactly 90°, where O denotes the center of the plant pot. Within the camera setup, meticulous arrangements are made to ensure that P1 and O appear at the same horizontal *x*-coordinate in camera 1, while P2 and O are positioned at the same horizontal *x*-coordinate in camera 2. This meticulous alignment guarantees consistent spatial correspondence between the reference points and the center of the plant pot, enabling accurate orientation measurements in both cameras.

With this system setup, we calculate the 3D distances between pixels viewed in the image planes of each camera. Camera 1 provides *x*-axis and *z*-axis information in world coordinate. By measuring the planting pot and plant positions in both cameras as drawn in **Figure 2C**, we can calculate the ratio (XRATIO, ZRATIO1) to convert distances between pixels in an image plane of camera 1 (*x*-axis, *z*-axis) to millimeters distance in world coordinate. We manually measure the width of plant pot (plant_pot_width) and choose the two corresponding points on ROI1 to calculate XRATIO (Equation 1). The distance between ground and the first leaf (ground_to_first_leaf) of the plant is measured to get ZRATIO1 (Equation 2).


(1) $$XRATIO=\frac{plant\_pot\_width}{\left|902-458\right|}=\frac{28}{444}=0.063.$$

(2) $$ZRATIO1=\frac{ground\_to\_first\_leaf}{\left|1345-756\right|}=\frac{63.1}{589}=0.107$$

Camera 2 provides *y*-axis and *z*-axis information of world coordinate. Similar to processing with camera 1, we manually measure the distance between two horizontal lines (dist_two_line) on the plant pot and their corresponding points on the image plane of camera 2. Then, YRATIO and ZRATIO2 to convert pixels in the image plane of camera 2 to millimeters in world coordinate can be calculated from Equations 3 and 4.


(3) $$YRATIO=\frac{plant\_pot\_width}{\left|973-519\right|}=\frac{28}{454}=0.0617$$

(4) $$ZRATIO2=\frac{dist\_two\_line}{\left|1644-1478\right|}=\frac{18}{166}=0.1084$$

Although we start recording videos from camera 1 (video 1) and from camera 2 (video 2) nearly the same time, the two videos are not perfectly synchronized. Based on voice channel in the videos, videos are cut into frames. Video 1 and video 2 have the same frame rate (30 fps), the same frame size (1,700 × 1,400 pixels), and the same starting time. The frames from each video are analyzed to identify the images of any insect in each frame and ultimately specify their 3D locations.

### 2.2 Deep learning-based insect detector

**Figure 4:**
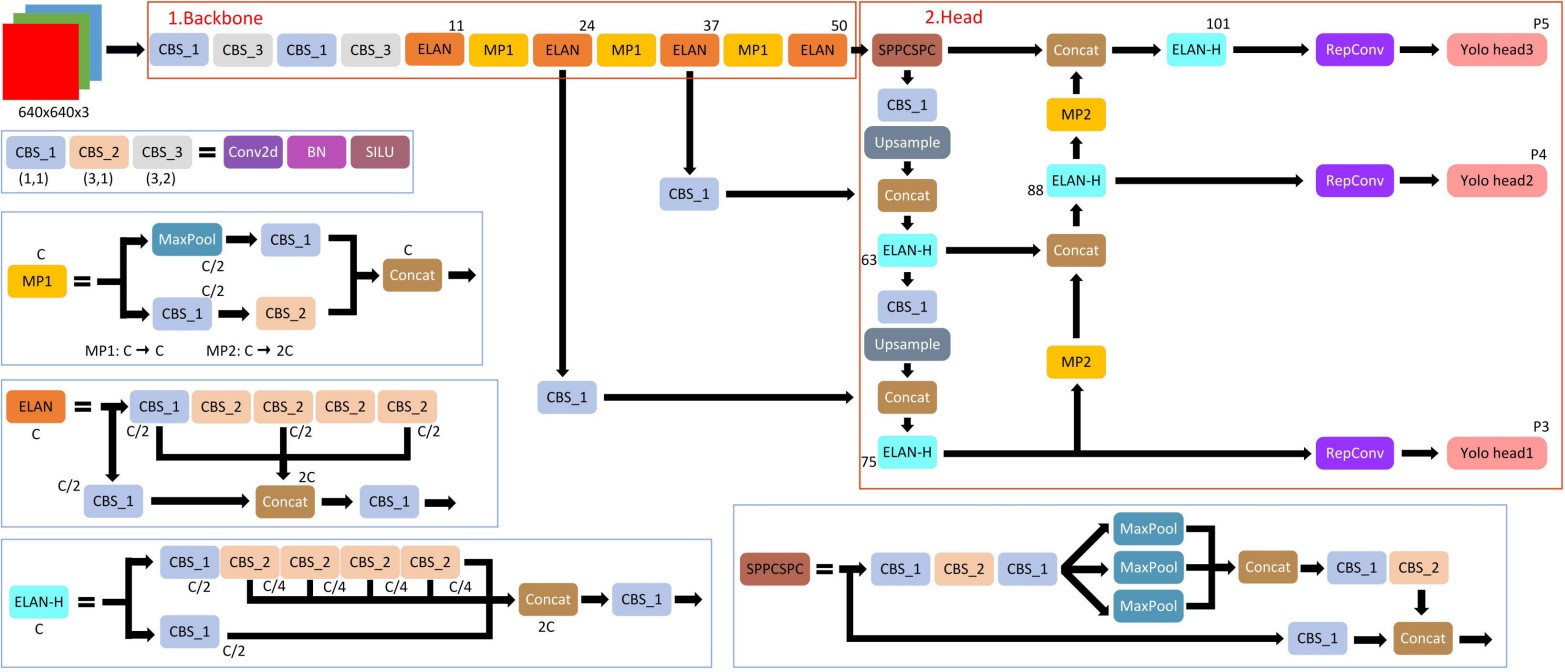
The structure of YOLOv7.

Recently, a state-of-the-art, real-time object detection algorithm was developed by Wang et al. (2023), namely, You Only Look Once version7 (YOLOv7) (Wang et al., 2023). The most recent YOLO algorithm outperforms all earlier object detection algorithms and YOLO variants in terms of accuracy and speed. YOLOv7 achieves 2% greater accuracy at a significantly faster inference speed (509% faster) than the top Cascade-Mask R-CNN models. YOLO receives a 2D image as an input and then predicts bounding boxes with class probabilities for each object. The real-time object detection accuracy is significantly increased by YOLOv7 without raising the inference costs. YOLOv7 effectively outperforms other well-known object detectors by reducing approximately 40% of the parameters and 50% of the computation required for state-of-the-art, real-time object detections. YOLOv7 uses far less expensive computational hardware than other deep learning models. Without any pre-learned weights, it can be trained significantly more quickly on tiny datasets. A detail structure of YOLOv7 is presented by Zhu et al. (2022) as **Figure 4**.

The YOLOv7 framework comprises three principal components: the Backbone, the Head, and the Neck. The Backbone’s primary role is to extract crucial image features and relay them to the Head through the Neck. The Neck, in turn, gathers the feature maps generated by the Backbone and constructs feature pyramids. Lastly, the Head encompasses output layers responsible for the final detections. It is noteworthy to mention that YOLOv7 shares similarities with Scaled YOLOv4, which is an extension of YOLOv4, since both were authored by the same individuals. However, the YOLOv7 paper introduces significant changes, including Architectural Reforms, incorporating E-ELAN (Extended Efficient Layer Aggregation Network) and Model Scaling for Concatenation-based Models. Additionally, Trainable BoF (Bag of Freebies) has been incorporated, enabling data-driven alterations of the convolution process for re-labeling of signal features based on minimization of the loss, which is the mean squared error (MSE) of the differences between the bounding box image pixels and the expected image pixels, “ground truth” (Wang et al., 2023) for each image in a frame. Loss in the Backbone component (**Figure 4**) is termed coarse loss, and loss in the Head component is termed fine loss.

#### 2.2.1 Dataset collection and labeling

**Figure 5:**
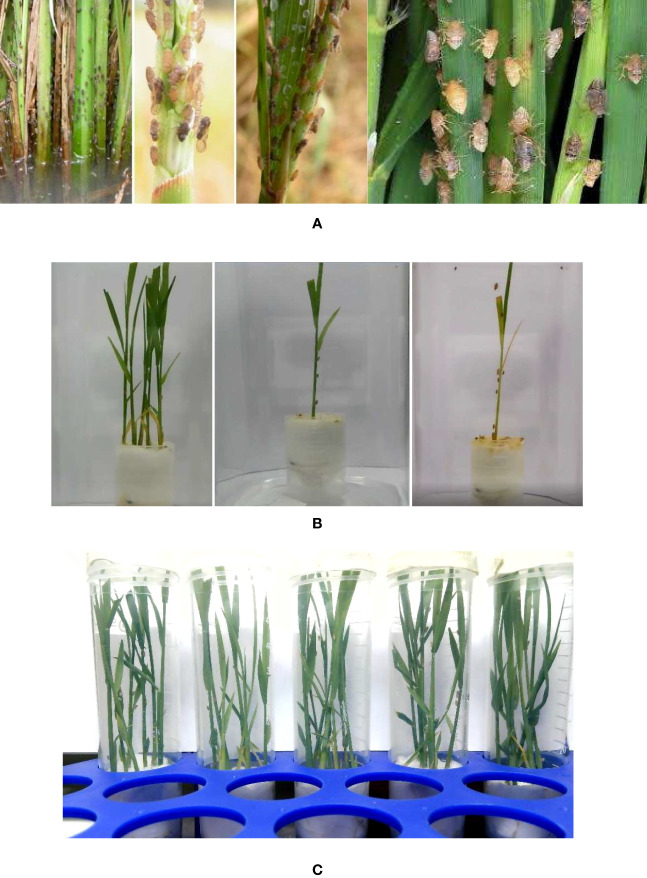
Images from our dataset. **(A)** Images that are downloaded from the internet contains brown hopper. Images that are captured for training. **(B)** From left to right: Image of insects with multiple plants, image of three insects on a single plant, and image of 10 insects on a single plant. **(C)** Image of multiple insects on multiple plants.

To build a dataset for training YOLOv7, we develop a Python script to characterize brown hopper crawling activities based on a series of internet images containing multiple insects, examples of which are shown in **Figure 5A**. After removing duplicate images irrelevant to the brown planthopper, *Nilaparvata lugens* (Stål) (Hemiptera: Delphacidae), we have a small-size dataset whose images contain multiple insects and multiple plants. The size of the bounding boxes for insects in those images varies, ranging from 12 × 15 pixels to 400 × 300 pixels. Following the acquisition of a small dataset of brown hopper images from the internet, we proceeded to establish a dedicated monitoring system for observing brown hopper behavior on rice plants. This system involved recording videos and capturing photos, and representative sample images are illustrated in **Figure 5C**. Concurrently, our monitoring system with dual cameras, as depicted in **Figure 1**, continuously captured videos of brown hoppers present on the rice plants featured in **Figure 5B**. We start with taking a video with one plant and no insect, then adding one insect after 2 h of taking the video. We capture the images from the videos as **Figures 5B, C**. The mean dimensions of the bounding boxes for insect amount to 12 × 17 pixels. Finally, we have a dataset with 594 images of insect in total, and we divide this into a training set and a testing set.

We use a labeling tool (‘Image annotation tool LabelImg’, 2023) to annotate the dataset. We draw a bounding box for each insect in each image. The bounding box must cover the entire insect and it should not include any extraneous background. If two insects are close together, try to avoid overlapping bounding boxes. Only create one bounding box for each insect in the image. All insects in the image should be labeled, especially those that are partially obscured.

#### 2.2.2 Training

After constructing the dataset, we train the deep neural network, YOLOv7, to detect individual brown planthoppers in frames. We train this with bounding box regression loss, object loss (insect loss), and classification loss. Bounding box regression loss measures the difference between the predicted bounding boxes and the ground truth bounding boxes for the insects in the image. It is calculated using the MSE loss. Insect loss measures the confidence of the model in predicting an insect in the image. It is calculated using the binary cross entropy (BCE) loss. Classification loss measures the difference between the predicted class probabilities and the ground truth class probabilities. It is also calculated using the BCE loss. The total loss used during training YOLOv7 is a weighted sum of the above losses, where the weights are determined empirically. Additionally, YOLO uses other techniques such as label smoothing and focal loss for further improving the training process.

**Figure 6:**
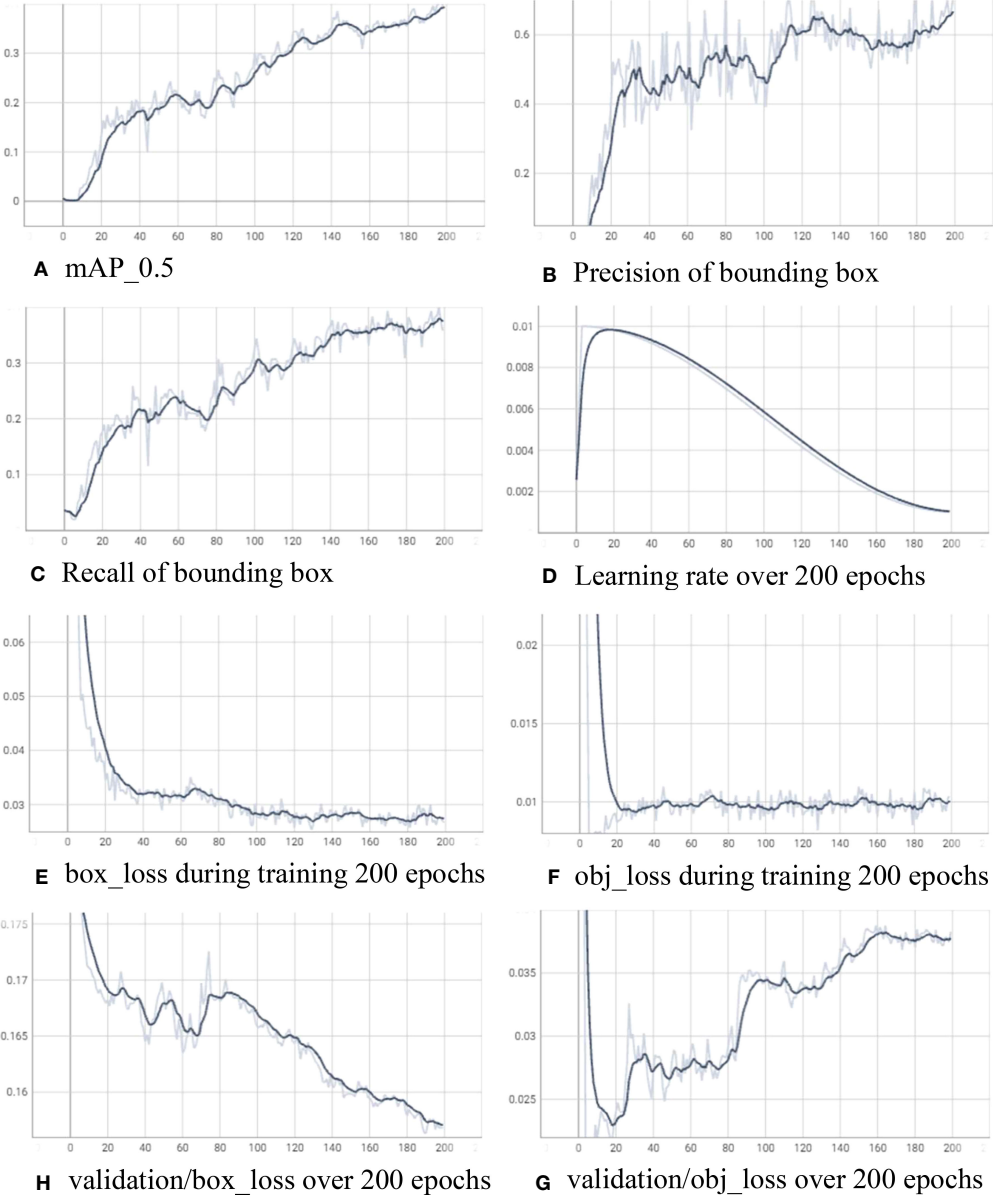
Training log for the insect detector with YOLOv7 over 200 epochs. **(A)** mAP_0.5, **(B)** precision of bounding box, **(C)** recall of bounding box, **(D)** learning rate over 200 epochs, **(E)** box_loss during training 200 epochs, **(F)** obj_loss during training 200 epochs, **(H)** validation/box_loss over 200 epochs, and **(G)** validation/obj_loss over 200 epochs.


For training the insect detector, we use a computer with an AMD Ryzen 7 5800x3D 8-core processor, RAM 32GB, and a graphic card NDVIA GeForce RTX 4080. In machine learning, an “epoch” refers to a single pass or iteration through the entire training dataset during the training process of the Yolov7. We train the model in 200 epochs with learning rate 0.01 and gradually decrease after warmup_epochs of 3. **Figure 6** shows the training log for YOLOv7 over 200 epochs. In **Figure 6A**, mean average precision (mAP) is evaluated during the training while precision and recall are presented in **Figures 6B, C**, respectively. The calculation of precision and recall is as follows:

(5) $$Precision=\frac{True\;positive}{True\;positive+False\;positive}$$

(6) $$Recall=\frac{True\;positive}{True\;positive+False\;negative}$$

where a true positive (TP) is the total number of correct positive predictions, meaning the model predicted that a pixel labeled as part of an insect image is correctly placed inside a bounding box. A false positive (FP) is the total number of predictions that the model predicts the pixel is inside the bounding box, but did not actually occur. A false negative (FN) is the total number of predictions that the model predicts the pixel is outside any bounding box, but actually did occur.

**Figure 6D** showcases the gradual decrease of the learning rate over the course of 200 epochs, with the final value reaching 0.00103. Simultaneously, the mean average precision at an intersection over union threshold of 0.5 (mAP_0.5) achieves a value of 0.3923, precision attains 0.7122, and recall reaches 0.3574. Throughout the training process, the box_loss and obj_loss on the training data consistently decrease, converging to 0.02661 and 0.01002, respectively. On the other hand, the box_loss on the validation data reaches 0.1568, while the obj_loss experiences a more rapid reduction after 20 epochs (reaching 0.02397) before slightly increasing to 0.03827 after 200 epochs.

#### 2.2.3 Detect 2D location of insect from image

After the insect detector is trained using the collected dataset through the above steps, for each frame in a video, we feed it into the insect detector to get 2D location of insect on the image. Then, the location is saved into a text label file. As a result, corresponding to a frame in a certain video, there is a corresponding label file. In this file, each detected insect in the image frame will have a label with five segments, including insect label (=0), center location (*x*_center_, *y*_center_) of the detected bounding box, and (width, height) of the detected bounding box.

### 2.3 3D insect location calculation using 2D locations

**Figure 7:**
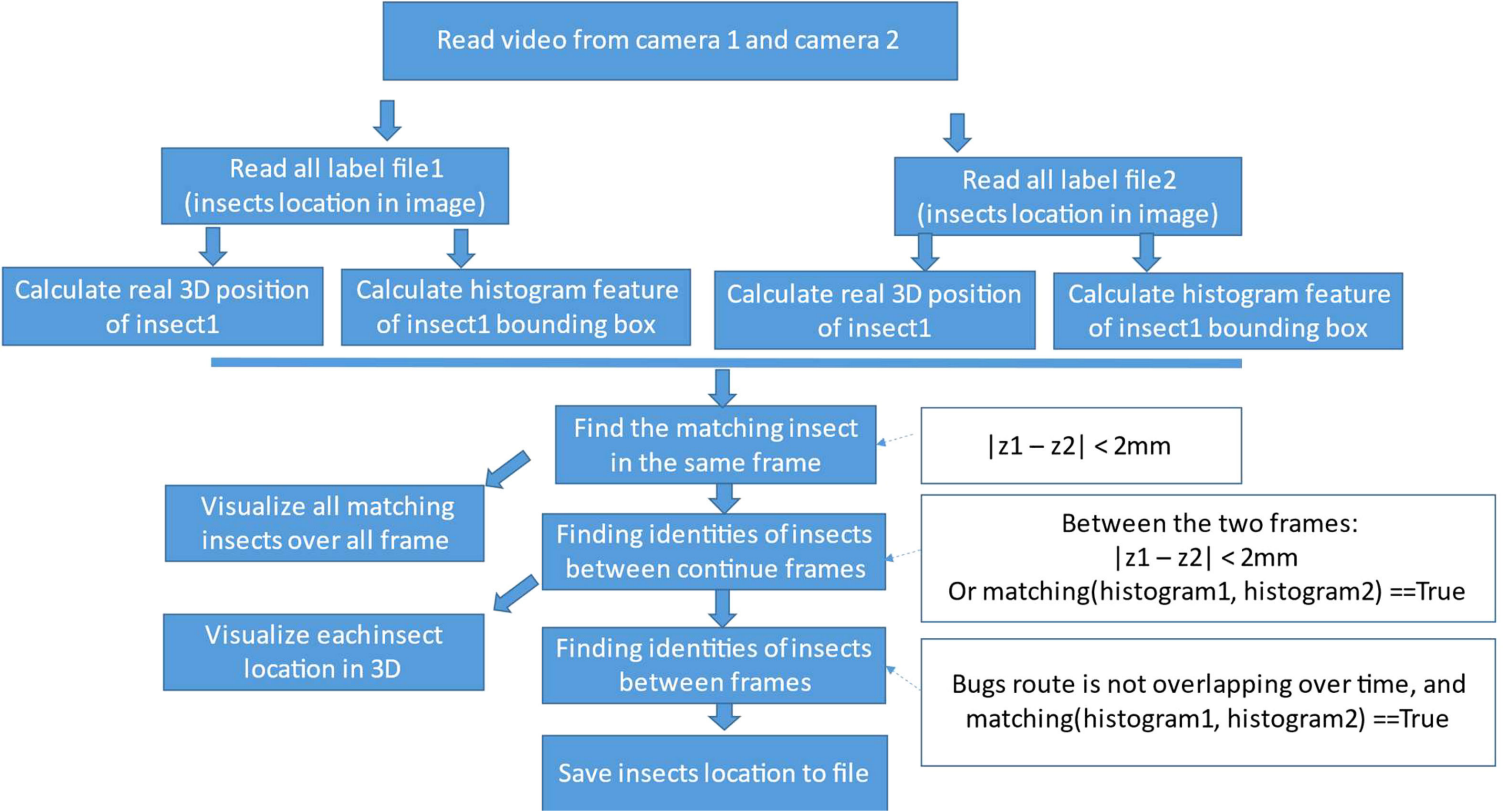
Flow diagram for calculation 3D insect location and visualization from 2D locations.

We run the trained insect detector on all recorded videos to get the 2D insect locations in them. **Figure 7** shows the flow diagram for getting 2D insect location from the label file to calculate 3D insect location and visualize the moving insect over frames in the two corresponding videos.

At first, we load two synchronized videos of camera 1 and camera 2 in the system and their list of insect locations inside each frame of the videos. For each bounding box of the detected insect, we resize the bounding box to the same size (HIST_W = 40, HIST_H = 40) and then calculate the color histogram of it as Equation 7. We use this as a matching feature for insect reidentification over frames in the video.


(7) $$h_{i}=\frac{n_{i}}{N}$$

where *h_i_* is the normalized value of the *i*th bin in the histogram, *n_i_* is the number of pixels with an intensity value equal to *i*, and *N* is the total number of pixels in the bounding box.

Based on the system specification, the position of each insect in 3D coordinates can be calculated using the 2D location of the insect on the two cameras. **Figure 1** shows one insect and its location in both cameras: camera 1 (*X*_1_ = 730, *Y*_1_ = 1278, *W*_1_ = 27, *H*_1_ = 35) and camera 2 (*X*_2_ = 745, *Y*_2_ = 1283, *W* = 22, *H*_2_ = 39), where *X*_1_, *Y*_1_ is the 2D location of the insect in the frame image captured from camera 1, and *W*_1_, *H*_1_ are width and height of the bounding box that cover the insect. Similarly, *X*_2_, *Y*_2_, *W*_2_, and *H*_2_ are bounding box information from camera 2.

We can calculate the real 3D location (*x*_real_, *y*_real_, *z*_real_) of insects by applying Equations 8, 10, 12, and 14 to pixel values and then converting them to millimeters using Equations 9, 11, 13, and 15. For *z*_real_, you can use camera 1(*z*_real1_) or camera 2 (*z*_read2_) to estimate.


(8) $$y_{realpixel}=X_{02}-X_{2}=722-745=-23\left(pixel\right)$$

(9) $$y_{real}=y_{realpixel}*YRATIO=-23*0.0617=-1.4191\left(mm\right)$$

(10) $$x_{realpixel}=X_{1}-X_{01}=730-737=-7\left(pixel\right)$$

(11) $$x_{real}=x_{realpixel}*XRATIO=-7*0.063=-0.441\left(mm\right)$$

(12) $$z_{realpixel1}=Y_{01}-Y_{1}=1350-1278=72\left(pixel\right)$$

(13) $$z_{real1}=z_{realpixel1}*ZRATIO1=72*0.108=8.667\left(mm\right)$$

(14) $$z_{realpixel2}=Y_{02}-Y_{2}=1364-1283=81\left(pixel\right)$$

(15) $$z_{real2}=z_{realpixel2}*ZRATIO2=81*0.107=8.667\left(mm\right)$$

As a result, from a list of videos from the two cameras, we separate each video into frames. In two frames at the same time from the two cameras, we use the insect detector to extract 2D locations of insects. Ultimately, a 3D representation of the position of each insect in the images is extracted from the 2D location calculations. Now, the insect needs to be tracked over frame. We use the relative distance between insect over the continuous frames and color histograms to track and reidentify the insects in 3D.

#### 2.3.1 Insect tracking and reidentification

In one frame t, there are some insects. Those insects are in the same locations on the next frame *t*+1 or they move to other locations or even disappear or partially disappear because of occlusion by the plant. Because the time between two continuous frames is small, approximately 1/30 s, the insect cannot move far from its location on the previous frame. In Equation 16, the $location\left(insects_{t,i}\right)$ is the location of the *i*th insect at frame *t*. The $distance_{frame}\left(\text{location }1,\text{location }2\right)\;$ is the distance between two points of location 1 and location 2 in 3D coordinates. From one frame *t*, with one insect *i* and its 3D real location $location\left(insects_{t,i}\right)$ , we find the nearest insect *j* among detected insects in the next continuous frame *t*+1 as Equation 16. If the distance between the two insects is smaller than threshold_distance_ (=2 mm in this paper) as in Equation 17, we consider them as the same insect in two continuous frames. Because of the small size of the insects, in some frames, the detector fails to detect insects correctly. Thus, based on the histogram and distance of the insects in different frames, we identify that the insects are newly appearing ones or they are identical.


(16) $$min_{j}=argmin_{j}\left(distance_{frame}\left(\mathrm{location}\left(\mathrm{insect}s_{t,i}\right),\mathrm{location}\left(\mathrm{insect}s_{t+1,j}\right)\right)\right)$$

(17) $$distance_{frame}\left(location\left(insects_{t,i}\right),\;location\left(insects_{t+1,min_{j}}\right)\right)<threshold_{distance}$$

(18) $$his_{diff}\left(t,i\right)=diff_{hist}\left(boundingbox\left(insects_{t,i}\right),boundingbox\left(insects_{t+1,min_{j}}\right)\right)$$

**Figure 8:**
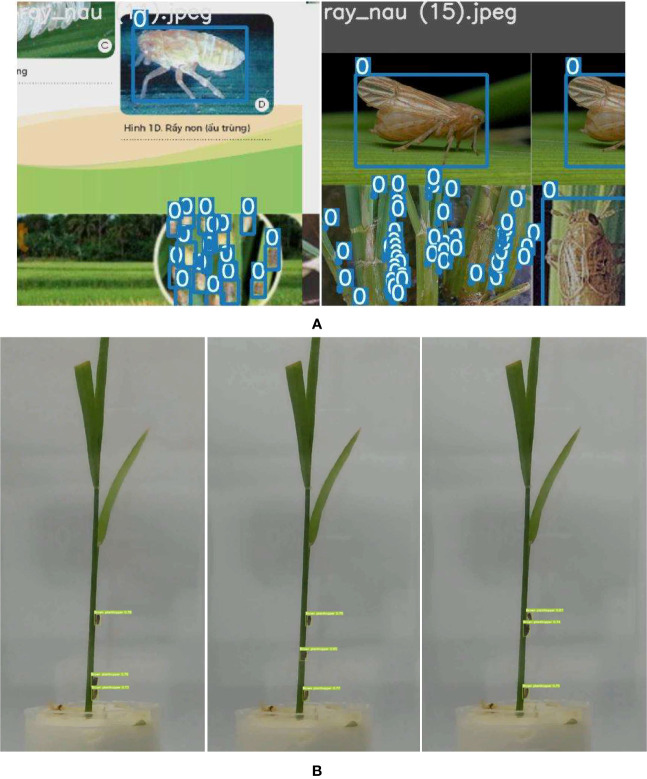
Detected insects on image using YOLOv7. **(A)** Detected insect during training on validation set. **(B)** Detected insect on testing video of three insects on a plant.

With the resized bounding box “ $boundingbox\left(insects_{t,i}\right)$ “ of detected insect *i* at frame *t*, the color histogram is calculated. The $diff_{hist}\left(box1,\;box2\right)$ is a function to calculate the difference between the histogram of box 1 and box 2. Histogram intersection is used to implement this function. This function calculates the area under the smaller histogram (minimum of corresponding bins) when both histograms are normalized to have the same sum (area under the curve). It measures the overlapping portion between the two histograms. Equation 18 is used for calculating the difference between the two histograms of the two bounding boxes of two given detected insects. For the *i*th insect in frame *t*, *insects_t+1,i_* is the insect *i* in the next frame *t* + 1. We find the *j*th *insect_t+1,j_* in the next frame of the frame *t*, which is the nearest to the *insect_t,i_* among detected insect in frame *t*+1 and $his_{diff}\left(t,i\right)\;$ is smaller than threshold_his_. Then, we consider that insect *i* in frame t and insect *j* in frame *t*+1 is the same insect. Then, we put the identified insect location into a list of location of the insect to visualize it as in **Figure 8**.

Furthermore, in some frames, the detector fails to detect the insects in one camera because of partial occlusion of insects. As a result, we fail to reidentify the insect between continuous frames. Then, a heuristic-approximation algorithm is applied that includes a “greedy” search component to enable a reidentification based on comparing the histogram of the bounding box of the frame in which an insect last appeared with histograms of the last appearing frame of other insects. The best match is selected to handle the reidentification process between non-adjacent frames. Consequently, we disregard the frames situated between the first and last frames, a characteristic that earns the method the designation of “greedy”. Our approach is underpinned by the observation that if one sequence contains insect *i* and another sequence partially overlaps with the sequence of insect *j*, then insect *j* is distinct from insect *i*. Leveraging this observation represents a pragmatic technique, which justifies our approach as heuristic. Furthermore, due to the presence of a noisy background, certain frames erroneously identify the background as insects within a brief duration. To mitigate this issue, we adopt a strategy to eliminate falsely detected insects by employing a frame count mechanism to assess the duration of insect appearance.

## 3 Results

### 3.1 Insect detection using YOLOv7

We feed frame by frame of the video to the insect detecting model to predict the bounding boxes of the insect in each frame. **Figure 8A** presents the detected bounding box on the validation after training. In each bounding box, there is a label (“0” in our case). **Figure 8B** presents the detected insect at the frame at the 25th, 42th, and 47th second in the testing video. There are three detected insects in each image frame. While only one insect (the insect in the middle) is moving up, the others keep the same location.

### 3.2 3D insect visualization

**Figure 9:**
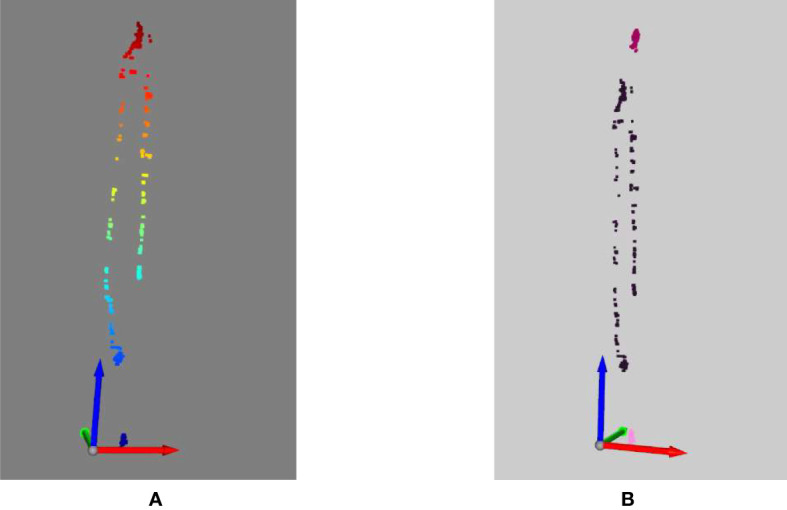
3D visualization of the insects. **(A)** No reidentification, **(B)** with reidentification.

For each frame of the video, the mplot3d toolkit (‘The mplot3d toolkit.’, 2023) is used to plot the sequence of the insect moving over time. In **Figure 9**, on the left side, we show all the locations of the insect over time. Different from the left side, on the right side of **Figure 8**, we show three identified insects in three different colors. The first insect is in dark pink color. It moved from the middle of the plant up to near the second insect (in purple pink color) and then moving down to the location of the third insect (in light pink color). In the video, the second and the third insect did not move much.

### 3.3 Statistical insect moving

Based on distances of the insect movement over time, we calculate the distance of the insect over frames. This distance gives information of the moving speed of the insect over frames. We use Euclidian distance (||.||_2_) to calculate the distance between the location of the *i*th insect in the frame *f* ( $location\left(insects_{f+1,i}\right)$ ) with its location in the previous frame ( $location\left(insects_{f,i}\right)$ ). Taking the sum over all frame of the video as Equation 19, we can get the moving distance over the whole video (moving_i_).


(19) $$moving_{i}=\sum_{f=1}^{numF-1}\|location\left(insects_{f+1,i}\right)-location\left(insects_{f,i}\right){\|}_{2}$$

**Figure 10:**
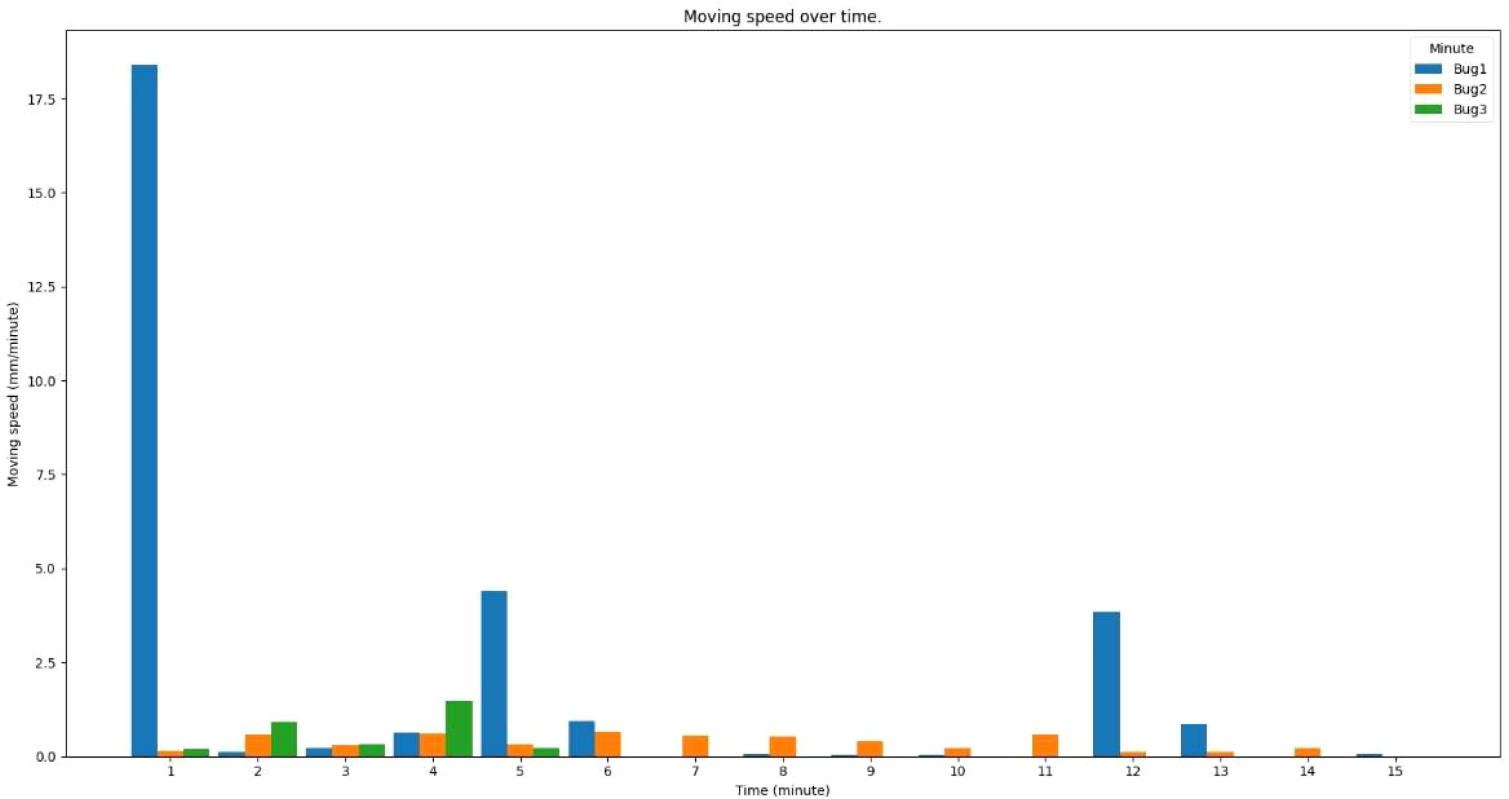
Moving speed of insects over time in the sample video.

**Figure 10** displays the movement speeds of three detected insects over a 15-min period. The first insect was moving approximately 18 mm in the first minute, 5 mm in the fifth minute, and 4mm in 12th minute in the recorded video, while other insects did not move much during the recorded period.

## 4 Discussion

The field of agriculture is currently experiencing a digital revolution, where there are a vast amount of data available at a relatively low cost of collection and transmission. Farmers are now faced with the challenge of analyzing “Big Data” using advanced algorithms to make decisions based on the interpretation, prediction, and inference of these data, potentially on a global scale. This involves organizing, aggregating, and interpreting the massive amount of available digital data to drive more informed decisions (Fan et al., 2014; Weersink et al., 2018). In addition, the implementation of computer vision science (Paul et al., 2020), machine learning (Liakos et al., 2018), deep learning (Kamilaris and Prenafeta-Boldú, 2018), and artificial intelligence (Jha et al., 2019) can reduce the cost of human interventions and maximize human output effectiveness.

Recent publications have highlighted practical applications of the Internet of Things (IoT) in monitoring and managing insect populations using camera-equipped traps. For example, crawling insects like cockroaches (Blattodea), beetle pests that infest stored food (Coleoptera), and ants (Hymenoptera: Formicidae) in urban environments have been monitored using IoT-based traps, as well as controlling the coffee berry borer (*Hypothenemus hampei* Ferrari), a pest of coffee crops. These applications demonstrate how IoT can be used to improve insect management by providing real-time data and insights to farmers and other stakeholders (Figueiredo et al., 2020; Preti et al., 2021).

The use of advanced technology in insect monitoring provides a unique opportunity to leverage expertise in 3D technology, electronics, informatics, and data analysis. By using two cameras, we were able to monitor the behavior and movement of insect with high precision. This camera-based system provides higher-quality images and videos compared to other automated systems that rely on infrared sensors. As a result, the insect can be checked with greater accuracy. This system is not limited to pest monitoring, but can also be used for early detection and surveying, particularly for invasive species. This enables quick responses to potential infestations and invasive species.

The use of camera-based monitoring can also aid in eradication programs for invasive species. Image processing algorithms can be employed to identify and automatically count insects, making observation easier. Image processing and computer vision techniques have been applied to automatically identify various insect pests, including the diamondback moth (*Plutella xylostella* L.), the Queensland fruit fly (*Bactrocera tryoni* Froggatt) (Liu et al., 2009), and the rice insect (*Leptocorisa chinensis* Dallas) (Fukatsu et al., 2012) (Fukatsu et al., 2012). For instance, Doitsidis et al. (2017) developed a detection and recognition algorithm using machine vision techniques to enable the automatic insect count of *B. oleae* (Doitsidis et al., 2017). These techniques can be applied to improve the effectiveness of eradication programs by providing accurate and real-time data to aid in decision-making.

Furthermore, owing to the excellent picture resolution that can be obtained by using our concept of 3D monitoring and the potential for wireless technology to be used for data transfer, it is now feasible to remotely control insect detection, behavior, and identification, hence minimizing the need for field trips. In addition to lowering the expenses associated with conventional field monitoring techniques, this offers the potential for enhanced pest control through more frequent and precise monitoring.

The utilization of a 3D monitoring system yields increased accuracy due to the inclusion of depth information. In contrast, conventional 2D monitoring systems only consider motion along the *x*-horizontal and *y*-vertical axes, omitting the *z*-depth dimension. This limitation becomes apparent when an insect moves parallel to the camera direction on a leaf. In a 2D system, such motion appears static, but a 3D system can discern the insect’s movement in the *z*-direction. The 3D system’s ability to detect motion in the depth dimension enhances its capacity to track insect movements more comprehensively, providing valuable insights in scenarios where *z*-direction motion is significant.

There are several practical applications that can be derived from the current system, which demonstrate the practical significance of the study. First, by systematically monitoring insects across a diverse range of plant species, this study provides valuable insights into their behavioral preferences. Consequently, it facilitates the identification of plant varieties that are less attractive to insects. This knowledge can significantly contribute to informed decision-making in agricultural practices, enabling the selection of plant species that exhibit greater resilience against insect infestations. Second, through the monitoring of insects during various treatment methodologies, it becomes possible to assess their responses. This evaluation process enables the identification of the most effective treatment options for preserving plant health and preventing insect-related damage. The outcomes of this research contribute to the development of efficient pest control strategies for farmers and agricultural researchers. Third, this study involves meticulous observation of how insects respond to changes in environmental conditions. By analyzing these responses, valuable insights into the adaptive mechanisms of insects can be gained. This knowledge is instrumental in predicting and managing insect behavior within different environmental contexts, thereby facilitating effective pest control measures and supporting ecosystem management efforts. Finally, simultaneously monitoring plants and insects during plant transformations, such as leaf discoloration, provides a unique opportunity to examine the intricate interplay between these phenomena and insect behavior. Through this investigation, valuable insights into the complex dynamics governing the interactions between plants and insects can be obtained. Such insights help unravel the underlying mechanisms that drive their interactions.

## 5 Limitation

The primary focus of this study lies in the monitoring of individual brown hopper insect on single plants, which limits the availability of sufficient data for evaluating insect reidentification. Given the current setup and the challenges posed by the blurriness of insect images with the current performance of insect reidentification, a drawback of our system is its inability to monitor a large number of insects simultaneously.

## 6 Conclusion

Using a 3D insect monitoring system, we were able to identify and detect insect movement and behavior. The 3D monitoring system is more accurate in visualizing the movement than the 2D system. The system can be used by entomologists and other researchers to study insect behavior and movement patterns. By providing detailed 3D visualizations of insects, the system can help researchers to better understand the ways in which insects interact with their environments. This system also cannot be limited to pest monitoring only; it can also be used for early detection and surveying of other invasive species, which enables quick responses to potential infestations. In summary, this 3D insect monitoring system can be a powerful tool for monitoring insect populations and controlling pest infestations, as well as for conducting research on insect behavior and ecology.

## Data availability statement

The original contributions presented in the study are included in the article/supplementary material. Further inquiries can be directed to the corresponding author.

## Author contributions

Conception and design: YC and NC. Data collection: KK, TT, and NC. Analysis and interpretation of results: TT, SM, and YC. Draft manuscript preparation: SM and TT. Manuscript review and validation: YC and NC. All authors contributed to the article and approved the submitted version.
